# Audience Design through Social Interaction during Group Discussion

**DOI:** 10.1371/journal.pone.0057211

**Published:** 2013-02-21

**Authors:** Shane L. Rogers, Nicolas Fay, Murray Maybery

**Affiliations:** School of Psychology, University of Western Australia, Perth, Western Australia, Australia; University of Lausanne, Switzerland

## Abstract

This paper contrasts two accounts of audience design during multiparty communication: audience design as a strategic individual-level message adjustment or as a non-strategic interaction-level message adjustment. Using a non-interactive communication task, Experiment 1 showed that people distinguish between messages designed for oneself and messages designed for another person; consistent with strategic message design, messages designed for another person/s were longer (number of words) than those designed for oneself. However, audience size did not affect message length (messages designed for different sized audiences were similar in length). Using an interactive communication task Experiment 2 showed that as group size increased so too did communicative effort (number of words exchanged between interlocutors). Consistent with a non-strategic account, as group members were added more social interaction was necessary to coordinate the group's collective situation model. Experiment 3 validates and extends the production measures used in Experiment 1 and 2 using a comprehension task. Taken together, our results indicate that audience design arises as a non-strategic outcome of social interaction during group discussion.

## Introduction

Audience design, or recipient design, is the process of speech adaptation to accommodate an addressee [1], [2]. While ‘design’ implies a thoughtful and strategic process of linguistic adjustment, it is equally possible that such adjustment is thoughtless and non-strategic (for a discussion of deliberative and non-deliberative thinking see [3]). When and the extent to which people consider the beliefs and knowledge of their audience during message design is a contentious issue [4], [5]. Fay, Garrod and Carletta [6] identified group size as a situational factor that promotes strategic audience design; speakers faced with larger audiences produced more informative messages (i.e., longer and more detailed messages that were easier for others to understand). This paper experimentally examines the extent to which people strategically design their message for different sized audiences, and contrasts this with an alternative account; that audience design arises as a non-strategic outgrowth of social interaction during group discussion. In other words, the behavioural studies reported here examine the extent to which audience design is a strategic, top-down and individual-level design process or a non-strategic, bottom-up and interaction-level process.

Classical theories of communication argue that audience design involves strategic adjustments in communication that are intentionally designed to meet the informational needs of one's addressee [7]–[9]. These theories postulate that perspective-taking plays a crucial part in the strategic design of successful messages; people consider their addressee's perspective (beliefs and knowledge) when constructing their message, and the feedback they receive allows them to update and refine this perspective. The development of a shared perspective, or situation model, allows interlocutors to reduce their collaborative effort (i.e., produce increasingly succinct messages that are tailored to their addressee's informational needs; [10]). On this account speakers have a model for specific addressees, and access this model when designing their message. This is supported by an empirical study showing that interlocutors develop ‘conceptual pacts’, addressee-specific agreements about how to label objects [11].

By contrast, non-strategic accounts claim that people pay little attention to the perspective of their addressee during language processing. Empirical studies indicate that speakers use particular syntactic structures to ease sentence production rather than to benefit addressee comprehension [12] and that addressees initially interpret utterances from their own perspective rather than taking the speaker's perspective [13]. The interactive alignment model [14] offers an alternative non-strategic account of how ‘conceptual pacts’ might arise. On this account, interlocutors adopt the same labels (and other aspects of linguistic representation, including prosody and syntax) as their partner via priming processes that operate during conversation. Linguistic representations produced by the speaker automatically activate similar representations in the addressee, and these representations retain enough activation such that when it is the addressee's turn to speak they are reused (and readily understood by the previous speaker). Unlike the strategic and computationally costly classical theories, the interactive alignment account stresses the role of non-strategic and computationally cheap priming.

The interactive alignment model argues that linguistic entrainment is automatic and implicit, a non-strategic outcome of interactive priming between interlocutors. However, and consistent with classical theories, beliefs about one's addressee can mediate linguistic entrainment. For example, stronger lexical entrainment is observed when participants believe they are playing a picture naming game with a computer as opposed to a human [15]. Even stronger entrainment is observed when participants believe they are interacting with a less capable computer, suggesting that beliefs about an addressee's communicative capacity can affect alignment in dialogue. Similarly, Kingsbury [16] showed that responses to requests for directions were longer and more detailed when the requester was perceived as being from out-of-town rather than a local. Similar strategic addressee-specific adjustments are seen in expert-layperson dialogue, indicating that beliefs about the expertise of one's addressee can affect message design [17].

Another situational factor that can affect message design is group size. Naïve overhearers are found to better understand what was agreed during ten-person group discussions when compared to smaller five-person discussions [6]. Fay et al [6] reasoned that speakers in the larger discussion groups were more sensitive to their broader audience, and engaged in more thorough message design to ensure the greater variety of perspectives contained in the larger group were catered to. This is consistent with audience design as a strategic individual-level process. However, and as noted by Fay et al, the dynamics of the different sized discussions were very different; small group discussions were more interactive than large group discussions (characterised by more frequent speaker switching, shorter speaker turns and more interruptions). Thus, an alternative hypothesis is that overhearers' better understanding of what was agreed during the larger group discussions arose not because of strategic adjustments during message formulation, but arose instead on account of the different communication dynamics prevalent in small and large group discussions. For instance, it is quite possible that overhearers' better comprehension of what was agreed in the larger group discussions resulted from hearing a broader range of perspectives on the topic under discussion. This competing explanation is consistent with audience design as a non-strategic interaction-level process.

The experiments reported here try to tease apart these competing explanations of audience design during multiparty communication. Using a non-interactive referential communication task, Experiment 1 tests if participants exert more effort (i.e., produce longer messages) when communicating to a larger group. By using a non-interactive task, Experiment 1 controls for multiparty communication dynamics, allowing for a test of the extent to which audience design reflects strategic individual-level message design. Experiment 2 uses an interactive referential communication task to study the effect of multiparty communication dynamics on audience design. This allows us to test if non-strategic interaction-level processes can explain the pattern of results observed by Fay et al [6]. Experiment 3, using an overhearer-type paradigm, validates and extends the results of Experiments 1 and 2.

## Experiment 1

To test if people strategically design their messages to meet the informational needs of their audience Fussell and Krauss asked participants to write descriptions of a range of abstract shapes where the intended audience was either themself or a stranger [18], or a friend or a stranger [19]. As predicted, messages designed for another person were longer (in words) and more accurately decoded by strangers compared to messages designed for oneself (which included more idiosyncratic shape descriptions). Furthermore, messages designed for a friend (i.e., a specific other) were more accurately decoded by that friend when compared to a stranger (i.e., a generic other). These findings indicate that people can strategically design their messages to meet the informational needs of their audience.

Experiment 1 extends these studies to determine if people make similar strategic message adjustments based upon their size of their target audience. Like Fussell and Krauss [18], [19] a non-interactive task is used in which participants write descriptions for a range of abstract geometric shapes. Audience size is manipulated by telling participants that their descriptions are for themself (Self), or for One, Four, or Nine other people (i.e., a 2-Person, 5-Person or 10-Person group, including the participant). Like Fussell and Krauss [18] participants were not given any specific information about the audience aside from its size. Thus, in the context of Experiment 1, strategic audience design is a broad adjustment in response to audience size as opposed to individually tailored addressee-specific message adjustments. As discussed, a non-interactive task is used to eliminate interaction-level processes.

If audience size is a strategic consideration during message design, then participants will exert more communicative effort when producing messages for larger audiences (as the number of potentially differing perspectives in the group increases). This will be reflected by longer messages (in words) as group size increases. This pattern of results would support audience design as a strategic individual-level adjustment.

## Method

### Participants

One hundred undergraduate students from the University of Western Australia participated in exchange for partial course credit or payment. All were native English speakers.

### Stimuli

**Figure 1:**
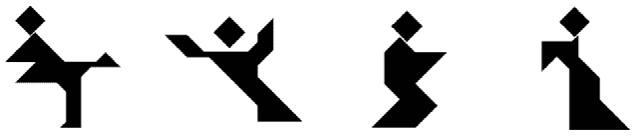
Example geometric shapes used as stimuli in the current study.

Eighteen abstract geometric shapes were used as stimuli (4 example shapes are given in Figure 1). This type of stimuli is frequently used to study language processes (e.g., [10], [20]–[23]).

### Procedure

Participants were randomly allocated to one of four conditions, with 25 participants in each. In each condition participants (henceforth referred to as ‘directors’ using Clark and Wilkes-Gibbs' terminology; [10]) wrote descriptions into a Microsoft word document for each of the 18 geometric shapes (presented within the same Microsoft Word document) for either Self, One, Four or Nine other people (i.e., Self, 2-Person, 5-Person or 10-Person group). Directors in the *Self* condition were instructed to write a description for each shape that would allow them to identify the target shape from the description. Directors in the *Other* conditions were asked to write descriptions that would allow One, Four or Nine others to pick out the intended geometric shape from its description. In each condition directors completed the task four times, with the array of shapes presented in a different random order on each game. This allowed us to examine the extent to which descriptions changed (e.g., became more succinct) over repeated reference.

## Results and Discussion

**Figure 2:**
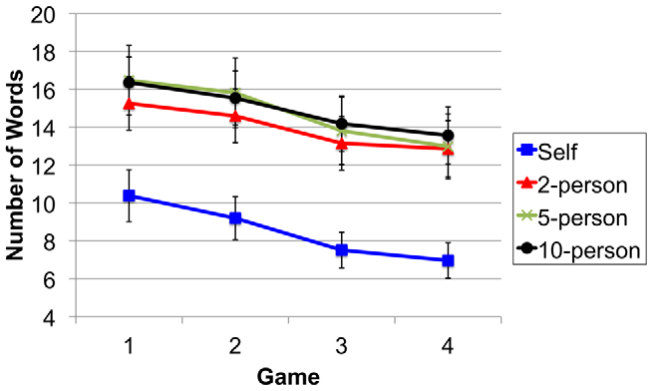
Mean number of words per shape, for each non-interactive condition across Games 1–4. Error bars indicate the standard errors of the means.

Message length (in words) was used to measure communicative effort. This is a standard measure of audience design, where longer messages indicate more strategic message adjustments [18], [19], [24]–[26]. Directors used shorter messages when the intended recipient was oneself compared to when the message was designed for another person/s. Audience size did not affect message length; messages designed for One, Four or Nine others were of a similar length. In each condition messages became increasing succinct over repeated reference (see Figure 2). These observations were confirmed by ANOVA.

Message length (in words) was entered into a mixed design ANOVA that treated Group Size (Self, 2-Person, 5-Person, 10-Person Group) as a between-participant factor and Game (1–4) as within. This returned a main effect of Group Size [*F*(3,96) = 4.76, *p*<.01, *n^2^* = .13] and Game [*F*(3,288) = 30.19, *p*<.01, *n^2^* = .24]. The Group Size by Game interaction was not significant (*p* = .98). The main effect of Group Size results from those in the Self condition using shorter messages to describe each shape compared to those in the other conditions (between-participant *t*s(48)>3.14, *p*s<.01, *d*s>.89). There was no difference in the length of messages used to describe each shape in the 2-Person, 5-Person and 10-Person Group Size conditions (*p*s>.63). Each level of Group Size showed a reliable decrease in message length across Games [one-way within-participant ANOVAs, *F*s(3,22)>3.10, *p*s<.05, *n^2^s*>.28].

This study asked if group size is a strategic individual-level consideration when constructing a message. Controlling for interaction-level processes, our results indicate that participants distinguished between the self and other; messages for another person/s were longer than those designed for oneself. This finding replicates Fussell and Krauss [18]. However, group size did not affect message length; messages for different sized audiences were of a similar length. Our results suggest that participants made strategic individual-level message adjustments based upon whether the destination of their message was themself or someone else. Contrary to Fay et al [6] this did not extend to different sized audiences (where messages were of a comparable length). The finding that participants reduced their communicative effort over repeated reference is consistent with other referential communication studies [10], [21], [27]–[29].

Controlling for interaction-level processes, Experiment 1 found no evidence to indicate that group size affects strategic audience design. By using an interactive referential communication task, Experiment 2 examines the effect of non-strategic interaction-level processes on audience design during group discussion.

## Experiment 2

Larger groups tend to contain a greater diversity of perspectives compared to smaller groups. The increase in the potential number of knowledge discrepancies increases the coordination problem in larger groups [30]. Consequently, members of larger groups tend to work harder to achieve mutual consensus, or coordinate their collective situation model [31]. The more elaborate coordination process in larger groups may have benefitted the overhearers in the Fay et al [6] study. With more perspectives to coordinate (or knowledge discrepancies to reconcile), it is likely that non-active group members are exposed to more perspectives on the topic under discussion. For instance, in the current task larger groups may negotiate several possible ways to describe each shape (e.g., the leftmost shape in Figure 1 was referred to as the “ice skater”, “ballerina”, “T man”, “piranha fish” by different participants) before a particular description type was accepted.

Experiment 2 examines non-strategic audience design using an interactive referential communication task. Experiment 2 asks if the greater knowledge discrepancy typical of larger groups requires more extensive social coordination. Participants communicated with other members of their group (2-Person, 3-Person or 5-Person) using a text chat tool that allowed them to interact in writing with their addressee/s over a computer network.

## Method

### Participants

Two hundred and fifty undergraduate students from the University of Western Australia participated in exchange for partial course credit or payment. All were native English speakers. None of the participants in Experiment 2 took part in Experiment 1.

### Stimuli

The same geometric shapes used in Experiment 1 were used in Experiment 2.

### Procedure

Participants were randomly assigned to one of three conditions: 2-Person group (25 groups, N = 50), 3-Person group (25 groups, N = 75) or 5-Person group (25 groups, N = 125). Participants were randomly assigned to the director or matcher role (where the matcher tries to identify each shape from its description). Each participant was seated at a separate computer terminal and all communication took place using an Internet text-based chat program (http://xchat.org/). The directors' goal was to successfully communicate each target shape, in writing, to each of the members of their group. Their text appeared on the matcher/s screen after the director pressed the return key. The matcher/s could freely interact with the director and each of the other matchers using the text chat program. All communication was available to all members of the group via a shared chat screen. The same set of 18 geometric shapes used in Experiment 1 was used in Experiment 2. In each condition participants completed the task four times, with the array of geometric shapes presented in a different random order on each game. Each trial ended when each matcher typed ‘Got it’ into the text-chat editor. Participant roles (director, matcher) were fixed throughout the experiment.

## Results and Discussion

Number of words again provided a measure of communicative effort. Two sets of analyses were conducted. The first assessed the extent to which the directors' first description for each shape was influenced by strategic individual-level audience size. The second assessed the extent to which the directors' shape descriptions were influenced by multiparty communication dynamics (i.e., non-strategic interaction-level audience design).

The artificiality of Experiment 1 may have diminished our findings; directors may have been unwilling to engage in extensive strategic audience design given the absence of their imagined audience. In Experiment 2 the audience was co-present and visible, participants seated at computer terminals in the same testing room. Sending a message with the text chat tool required the director to press the return key. Thus, the first message produced by the director for each shape at Game 1 occurred in the absence of matcher feedback (i.e., it is non-interactive). With a co-present audience, did the directors produce longer messages for larger audiences? Again, the answer is no; message length was similar across the 2-Person (*M* = 13.79 words, SD = 7.17), 3-Person (*M* = 12.03 words, SD = 6.06) and 5-Person groups (M = 10.21, SD = 7.70; one-way between-participant ANOVA, *p* = .20).

Did the co-present audience promote more strategic audience design relative to an absent audience? Comparing the first message produced by directors for each shape in Experiments 1 and 2 indicated that participants produced shorter messages when their audience was co-present (compared across equivalent 2-Person and 5-Person groups). This was confirmed by a 2×2 ANOVA treating Condition (Experiment 1 Non-interactive, Experiment 2 Interactive) and Group Size (2-Person, 5-Person) as between-participant factors. The ANOVA returned a main effect of Condition [*F*(1,96) = 6.12, *p*<.05, *n^2^* = .06], indicating that participants produced shorter messages in the interactive context (compared to the non-interactive context). No other effects reached statistical significance (*p*s>.13). In fact, the mean length of directors' first message for each shape in the interactive context (Experiment 2) did not differ from the length of messages produced for oneself in the non-interactive context (Experiment 1; one-way between-participant ANOVA, *p* = .24).

In summary, group size did not affect strategic audience design in the interactive context. In fact, participants engaged in strategic audience design less in the interactive context (Experiment 2) when compared to the non-interactive context (Experiment 1). Compared to the non-interactive context, participants in the interactive context appear to minimize their communicative effort by offering a more succinct message at the outset that can later be refashioned based upon addressee feedback if need be.

**Figure 3:**
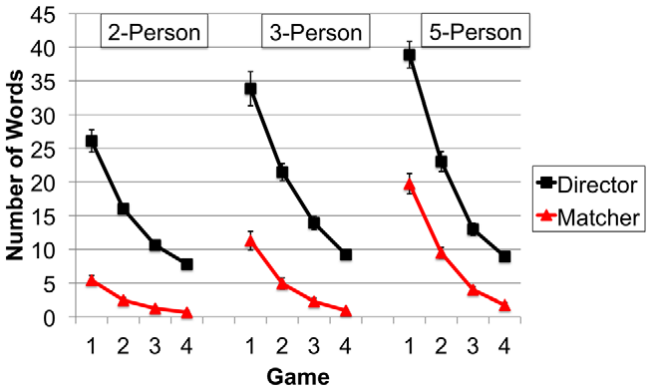
Mean total number of words produced by directors and matchers to communicate each geometric shape across Games 1–4 when in a 2-Person, 3-Person or 5-Person Group. Error bars indicate the standard errors of the means.

The next set of analyses examines the extent to which audience design results from non-strategic interaction-level processes. As group size increased so too did the mean total number of words produced by each director to describe each shape (Total mean number of words is the total number of words produced by the director to describe each of the geometric shapes divided by the total number of shapes. This calculation was conducted separately for each director and for each set of shape descriptions at Game 1 to Game 4.). The same pattern was observed among matchers; the mean total number of words produced by each matcher increased as group size increased. Over games 1 to 4, the mean total number of words produced by directors and matchers decreased (see Figure 3). These observations were confirmed by ANOVA.

The mean total number of words contributed by each director was entered into a mixed design ANOVA that treated Group Size (2-Person, 3-Person, 5-Person) as a between-participant factor and Game (1–4) as within. This returned a main effect of Group Size [*F*(2,72) = 9.73, *p*<.01, *n^2^* = .21] and Game [*F*(3,216) = 335.30, *p*<.01, *n^2^* = .82], in addition to a Group Size by Game interaction [*F*(6,216) = 6.87, *p*<.01, *n^2^* = .16]. The interaction is explained by the greater number of words produced by directors in the larger groups at Game 1 [5-Person = 3-Person, between-participant *t*(48) = 1.58, *p* = .12, >2-Person, *t*s(48)>2.57, *p*s<.05, *ds*>.74], and the comparable number of words produced by directors in the different sized groups at Game 4 (*p*s>.12). A similar pattern is observed when the mean total number of matcher words are entered into the same ANOVA: a main effect of Group Size [*F*(2,72) = 35.44, *p*<.01, *n^2^* = .50] and Game [*F*(3,216) = 178.30, *p*<.01, *n^2^* = .71], in addition to a Group Size by Game interaction [*F*(6,216) = 21.87, *p*<.01, *n^2^* = .38]. Like directors, the interaction is explained by matchers producing more words in larger groups at Game 1 [5-Person>3-Person>2-Person, *t*s(48)>3.73, *ps*<.01, *d*s>1.12], and a smaller difference at Game 4 [*p*s>.06, with exception that 5-Person>2-Person; between-participant *t*(48) = 2.41, *p*<.05, *d* = .69].

Consistent with a non-strategic account, these findings indicate that as group size is increased more effort is required (by directors and matchers) to coordinate the groups' collective situation model. Contrary to a strategic account, the greater communication burden is placed on matchers rather than directors. Matchers' relative contribution (total words contributed by matchers divided by total words contributed by directors and matchers) increased as group size increased from 2- to 3- to 5-Persons (16.45%, 24.17% and 33.18% at Game 1, and 6.52%, 8.91% and 14.82% at Game 4). The same pattern of results for director words and matcher words is returned when these percentage scores are entered into the same mixed design ANOVA. Again there is a main effect of Group Size [*F*(2,72) = 19.92, *p*<.01, *n^2^* = .36] and Game [*F*(3,216) = 114.86, *p*<.01, *n^2^* = .62], in addition to a Group Size by Game interaction [*F*(6,216) = 3.54, *p*<.01, *n^2^* = .10]. The interaction is explained by the greater percentage of words contributed by matchers in the larger groups at Game 1 [5-Person>3-Person>2-Person, between-participants *t*s(48)>3.02, *p*s<.05, *ds*>.85] and a smaller difference at Game 4 [*p*s>.07 with exception that 5-Person>2-Person; between-participant *t*(48) = 2.64, *p*<.05, *d* = .76].

Interaction-level processes had a strong effect on communicative effort; directors produced more words as a result of their interactions with matchers, and as more matchers were added (with potentially different perspectives) more social interaction was necessary to communicate the different shapes (driven primarily by the matchers). Evidence for this is reflected by a positive correlation between matcher words and director words at Game 1 [*r*(75) = .59, *p*<.01], Game 2 [*r*(75) = .49, *p*<.01] and Game 3 [*r*(75) = .39, *p*<.01]. With so few words produced by directors and matchers at Game 4, the correlation did not reach statistical significance [*r*(75) = .14, *p* = .23].

Using an interactive communication task Experiment 2 showed that group size affects communicative effort. This arose on account of non-strategic interaction-level processes rather than strategic individual-level message design. In fact, directors behaved more egocentrically (initial messages) when the audience was co-present (compared to absent). Director and matcher behaviour (words contributed) was interdependent; as more matchers were added more social interaction was needed to establish a shared situation model.

## Experiment 3

In Experiment 1 and 2 number of words produced was used as a proxy for audience design, where more words equated to more audience design. While number of words has often been used in this way, a problem with this measure is that it says nothing about the content of the message. It is quite possible that two descriptions that are comparable in length may differ in terms of message informativeness. For example, one description might repeat a description from the same perspective whereas the other might add an additional perspective on the same shape. Experiment 3 sought to validate and extend the results of Experiment 1 and 2 by examining naïve participants' ability to pick out the intended shape from its description. This comprehension score complements the production scores used in Experiment 1 and 2.

Actively participating in a conversation yields superior comprehension compared to passively overhearing the same conversation [21]. However, some types of non-participation are better than others. Fox Tree [22] compared overhearerer's comprehension when listening in on a two-party dialogue or a single-party monologue (where speakers described a range of abstract geometric shapes from an array). Comprehension was higher when overhearing a two-party dialogue. A follow-up study indicated that this comprehension benefit arose on account of being exposed to multiple perspectives in the dialogue condition, but only a single-perspective description in the monologue condition [32].

Experiment 3 extends these studies to determine if non-active participants will show a comprehension benefit as the number of perspectives is increased from one to two to many (as is likely to be the case in the context of multiparty communication). The written descriptions produced by participants in Experiment 1 and 2 were given to naïve participants who tried to pick out the target shape from its description. Our first prediction is that Experiment 1 descriptions that are designed for another person/s will be more accurately identified than those designed for oneself (as per [18]). This would suggest that the strategic message adjustments made by directors (indicated by an increase in message length) were successful. Given their comparable message length, no difference in identification rates is expected between the messages designed for different sized audiences in Experiment 1. Experiment 2 descriptions will return higher comprehension scores when compared to the Experiment 1 descriptions. This prediction is made on account of the longer descriptions in Experiment 2 and the greater number of perspectives they are likely to contain. Our final prediction is that descriptions produced in the larger interactive groups in Experiment 2 will be associated with improved comprehension. This prediction is again made on account of the longer descriptions and greater number of perspectives they are likely to contain.

## Method

### Participants

One hundred and seventy-five undergraduate students from the University of Western Australia participated in exchange for partial course credit or payment. All were native English speakers. None of the participants in Experiment 3 took part in Experiment 1 or 2.

### Stimuli

The geometric shapes and descriptions produced by participants in Experiment 1 and 2.

### Procedure

Participants were randomly assigned to one of 7 conditions: Self, 2-Person, 5-Person or 10-Person descriptions (Experiment 1), 2-Person, 3-Person or 5-Person descriptions (Experiment 2). Twenty-five participants were assigned to each condition. Experiment 3 participants were given an A4 sheet of paper containing each of the 18 shapes (presented in a different random order for each participant). They were also given the shape descriptions from the relevant condition (single speaker descriptions from Experiment 1 or an interactive transcript from Experiment 2) and asked to match the shape to the description (by inserting the shape number next to the description). Shape descriptions were given in the same order participants in Experiment 1 and 2 produced them. Only shape descriptions at Game 1 were used.

## Results and Discussion

**Figure 4:**
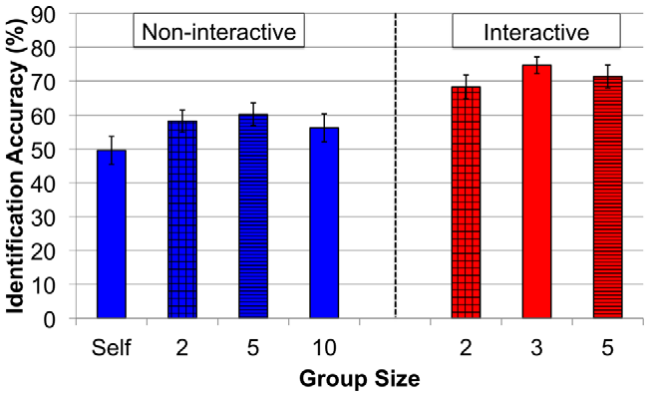
Naïve participants' comprehension (% correctly identified) of descriptions produced under the different conditions of Experiment 1 and Experiment 2. Error bars indicate the standard errors of the means.

We first examined participants' ability to pick out the target shapes from the descriptions given by directors in Experiment 1 (non-interactive). Comprehension of messages produced for different sized groups (2-Person, 5-Person and 10-Person) in Experiment 1 were understood equally well (one-way between-participants ANOVA, *p* = .73). Messages designed for another person (collapsed across the 2-Person, 5-Person and 10-Person conditions) were better identified than messages designed for oneself (between-participant *t*(98) = 2.08, *p*<.05, *d* = .49). Next we examined participants' ability to pick out the intended shapes from the interactive communication between directors and matchers in Experiment 2. Like Experiment 1, group size (2-Person, 3-Person, 5-Person) did not affect comprehension (one-way between-participants ANOVA, *p* = .36). Thus, the greater amount of words used in the larger interactive discussions did not translate into better comprehension by naive participants (see Figure 4).

The final analysis compares the comprehension of naïve participants across the non-interactive and interactive experiments. We compared the 2- and 5-Person groups from Experiment 1 (non-interactive) with the 2- and 5-Person groups from Experiment 2 (interactive). Comprehension scores were entered into a between-participant ANOVA with Group Size (2-Person, 5-Person) and Condition (Non-interactive, Interactive) as factors. This returned a main effect of Condition [*F*(1,96) = 9.80, *p*<.05, *n^2^* = .09], but no effect of Group Size or Group Size by Condition interaction (*p*s>.45). The main effect of Condition indicates that the interactive group discussions facilitated better comprehension among non-active participants (compared to non-interactive monologues).

## General Discussion

We contrast two accounts of audience design during multiparty communication: audience design as a strategic individual-level message adjustment, and audience design as a non-strategic interaction-level adjustment. Naïve overhearers' better understanding of what was agreed in larger group discussions led Fay et al [6] to conclude that members of larger groups engaged in more extensive strategic individual-level audience design to ensure the greater variety of perspectives contained in the larger group were catered to. However, Fay et al noted that the communication dynamics in the small and large groups were very different; small group discussions were more interactive than large group discussions. Thus, the observed comprehension benefits may have resulted from the different communication dynamics typical of small and large group discussions. This alternative explanation is consistent with audience design as a non-strategic interaction-level adjustment.

The behavioural studies reported try to tease apart the effects of strategic and non-strategic message design by examining audience design in a non-interactive and interactive multiparty communication task (Experiment 1 and 2 respectively). Controlling for interaction-level processes, Experiment 1 showed that messages designed for another person were longer (number of words) than those designed for oneself, a finding consistent with strategic message design. However, audience size did not affect message length (messages designed for One, Four or Nine others were similar in length), indicating that audience size does not affect strategic message design. Allowing for interaction-level processes, Experiment 2 showed that as group size increased so too did the number of words exchanged by the members of the group. However, this did not occur on account of strategic message design processes; directors' initial messages (prior to addressee feedback) in Experiment 2 (interactive) were shorter than those produced by directors in Experiment 1 (non-interactive) for the same sized audiences. With a co-present audience, directors seemed content to initially offer limited information, the meaning of which could later be interactively negotiated if necessary. The increase in communicative effort (number of words exchanged between group members) as group size increased in Experiment 2 suggests an important role for non-strategic interaction-level processes; as more addressees were added more social interaction was needed to negotiate a description of each shape that was acceptable to each group member.

Using an overhearer-type paradigm Experiment 3 extended the findings of Experiments 1 and 2. Longer messages that were designed for another person (as opposed to oneself; Experiment 1) were more informative; their intended audience understood them better (replicating [18]). Thus, participants' strategic individual-level message adjustments were rewarded by improved matcher comprehension. Furthermore, shape descriptions that were interactively negotiated (Experiment 2) contained more words than individually designed messages (Experiment 1) and were more accurately decoded by non-active participants. This finding supports a role for non-strategic message design, and replicates those showing the comprehension benefits of listening in on dialogues compared to monologues [22], [32], [33]. Contrary to expectations, the greater number of words exchanged in the larger interactive groups did not translate into improved comprehension by non-active participants in Experiment 3. One explanation is that number of words is not an accurate guide to the number of perspectives exchanged [32]. The increased number of words exchanged in the larger interactive groups may reflect greater perspective elaboration rather than the addition of unique perspectives. Another explanation is that it is the grounding process that benefits comprehension (rather than the number of unique perspectives; [33]). If correct, this would explain the broad difference in comprehension rates between the interactive and non-interactive contexts.

Taken together, the studies reported here indicate an important role for non-strategic processes to message design during group communication. While our study indicated a role for strategic individual-level audience design, this was only in so far as people distinguished between their own informational needs and the needs of someone else, and did not extend to different sized audiences (contrary to [6]). Consistent with a non-strategic interaction-level account, our participants did not consider audience size during initial message planning, but instead adjusted their messages on the fly, in response to feedback they received from their audience. These behavioural findings complement a recent online study (using reaction times to measure speech planning) that shows that message design is primarily a result of other-prompted rather than self-prompted speech adaptation [34]. Like this study, our findings indicate that audience design during multiparty communication is an adaption achieved through monitoring and adjustment during social interaction rather than being pre-planned at the individual-level.
